# Responses of branching reef corals *Acropora digitifera* and *Montipora digitata* to elevated temperature and pCO_2_

**DOI:** 10.7717/peerj.10562

**Published:** 2020-12-21

**Authors:** Cristiana Manullang, Intan Herwindra Millyaningrum, Akira Iguchi, Aika Miyagi, Yasuaki Tanaka, Yukihiro Nojiri, Kazuhiko Sakai

**Affiliations:** 1 Graduate School of Engineering and Science, University of the Ryukyus, Nishihara, Okinawa, Japan; 2 Geological Survey of Japan, National Institute of Advanced Industrial Science and Technology, Tsukuba, Ibaraki, Japan; 3 Department of Bioresources Engineering, National Institute of Technology, Okinawa College, Nago-City, Okinawa, Japan; 4 Environmental and Life Sciences, Universiti Brunei Darussalam, Brunei Darussalam; 5 Center for Global Environmental Research, National Institute for Environmental Studies, Tsukuba, Ibaraki, Japan; 6 Graduate School of Earth and Environmental Sciences, Hirosaki University, Hirosaki, Aomori, Japan; 7 Sesoko Station, Tropical Biosphere Research Center, University of the Ryukyus, Motobu, Okinawa, Japan

**Keywords:** Ocean acidification, Ocean warming, Corals, Calcification rate, Endosymbiont species

## Abstract

Anthropogenic emission of CO_2_ into the atmosphere has been increasing exponentially, causing ocean acidification (OA) and ocean warming (OW). The “business-as-usual” scenario predicts that the atmospheric concentration of CO_2_ may exceed 1,000 µatm and seawater temperature may increase by up to 3 °C by the end of the 21^st^ century. Increases in OA and OW may negatively affect the growth and survival of reef corals. In the present study, we separately examined the effects of OW and OA on the corals *Acropora digitifera* and *Montipora digitata*, which are dominant coral species occurring along the Ryukyu Archipelago, Japan, at three temperatures (28 °C, 30 °C, and 32 °C) and following four pCO_2_ treatments (400, 600, 800, and 1,000 µatm) in aquarium experiments. In the OW experiment, the calcification rate (*p* = 0.02), endosymbiont density, and maximum photosynthetic efficiency (*Fv/Fm*) (both *p* < 0.0001) decreased significantly at the highest temperature (32 °C) compared to those at the lower temperatures (28 °C and 30 °C) in both species. In the OA experiment, the calcification rate decreased significantly as pCO_2_ increased (*p* < 0.0001), whereas endosymbiont density, chlorophyll content, and *Fv/Fm* were not affected. The calcification rate of *A. digitifera* showed greater decreases from 30 °C to 32 °C than that of *M. digitata*. The calcification of the two species responded differently to OW and OA. These results suggest that *A. digitifera* is more sensitive to OW than *M. digitata*, whereas *M. digitata* is more sensitive to OA. Thus, differences in the sensitivity of the two coral species to OW and OA might be attributed to differences in the endosymbiont species and high calcification rates, respectively.

## Introduction

The rise in atmospheric CO_2_ concentration due to anthropogenic emissions is causing ocean warming (OW) and ocean acidification (OA). Atmospheric CO_2_ levels are predicted to increase from 400 to 1,000 µatm, and the global mean sea surface temperature is expected to increase by up to 3 °C and surface ocean pH to decrease by 0.3 units relative to 1986–2005 by the end of the 21st century under the “business as usual” emissions scenario (IPCC et al., in press).

Corals are the dominant marine calcifiers in coral reefs and play important roles in supporting coral reef ecosystems (*Knowlton, 2001; Wild et al., 2004*). Some studies have shown that the calcification rate of corals has declined because of OW in the last few decades (*Cooper et al., 2008; Tanzil et al., 2009; Cantin et al., 2010*). The optimal temperature for the growth and calcification of corals is generally close to the maximum summer temperature in ordinary years at a given reef (reviewed by Pratchett et al. (2015). When the temperature rises to 1 °C above the local summer maxima, the mutualistic relationship between the host coral and its symbiotic algae (unicellular algae of the family Symbiodiniaceae (*LaJeunesse et al., 2018*) or endosymbionts) is disrupted, with host corals losing their endosymbionts and becoming bleached (Glynn & D’croz, 1990; Glynn, 1996). OW suppresses the calcification of reef-building corals by affecting their endosymbionts (*Glynn, 1996; De’ath, Lough & Fabricius, 2009*), although moderate increases in seawater temperature facilitate coral calcification (*Inoue et al., 2012*). The endosymbionts provide energy to the host coral through photosynthesis (*Muscatine, McCloskey & Marian, 1981*). As the temperature rises above the bleaching threshold, endosymbiont density and photosynthesis decline (*Jones et al., 1998*), reducing the availability of the algal-derived photosynthate that fuels coral calcification (Al-Horani, Al-Moghrabi & De Beer, 2003). The optimal temperature for coral calcification may vary according to the temperature regime and location of the reefs (Marshall & Clode, 2004). Generally, maximum skeletal growth has been found to occur at a normal seawater temperature during the warm season at individual locations, and skeletal growth rates begin decreasing when the sea surface temperature rises above these temperatures (Jokiel & Coles, 1977; Coles & Jokiel, 1978; Marshall & Clode, 2004; Pratchett et al., 2015). *Acropora cervicornis*, from the Caribbean, was also reported to have maximum growth rates when the temperature ranged from 28 °C to 30 °C, and growth rates decreased at both higher and lower temperatures (*Shinn, 1966*). The calcification rates of *Acropora hyacinthus* and *Acropora muricata* decreased by 90% when exposed to temperatures 2.5 °C above the maximum summer temperature in the Great Barrier Reef (*Anderson et al., 2019*). In the Great Barrier Reef, the calcification rates of massive *Porites* declined by 11.4% from 1990 to 2005 because of reduced skeletal extension, which linearly correlated with an increase in the sea surface temperature (*De’ath, Lough & Fabricius, 2009*).

Responses to OW may differ among coral species (*Loya et al., 2001; Baird & Marshall, 2002; Bak, Nieuwland & Meesters, 2009; De’ath, Lough & Fabricius, 2009; Grottoli et al., 2018*). This might be because of differences in endosymbiont species (*Berkelmans & Van Oppen, 2006; Sampayo et al., 2008; LaJeunesse, Pettay & Sampayo, 2010*), as different species of endosymbionts have different tolerance levels to heat stress (*Fitt & Warner, 1995; Bhagooli & Hidaka, 2003; LaJeunesse et al., 2003; Berkelmans & Van Oppen, 2006; Fitt et al., 2009*) or provide different quantities of photosynthate (*Anthony et al., 2009; Cantin et al., 2009; Hughes & Grottoli, 2013; Schoepf et al., 2015; Tremblay et al., 2016*). *Berkelmans & Van Oppen (2006)* showed that colonies of *Acropora millepora* containing different genera of endosymbionts had different heat tolerances on the Great Barrier Reef. Differences in the species of endosymbiont within a genus may also affect the heat tolerance of corals. For example, *Fisher, Malme & Dove (2012)* reported that coral species hosting a given species of endosymbionts were more susceptible to heat stress compared to those hosting other species in the Great Barrier Reef.

As CO_2_ in the ocean reacts with seawater, it lowers the pH and shifts the carbonate equilibria, decreasing the carbonate ion concentration and lowering the calcium carbonate saturation state or Ω (*Millero, 1995; Caldeira & Wickett, 2003; Sabine et al., 2004; Feely, Doney & Cooley, 2009*). Decreasing Ω in seawater may not directly affect coral calcification because calcification occurs in calcifying fluid isolated from ambient seawater (*Cyronak, Schulz & Jokiel, 2016*). OA may reduce the calcification of corals by affecting the physiology of the endosymbionts and host corals (Hoegh-Guldberg et al., 2007; Anthony et al., 2008; Anthony, Kleypas & Gattuso, 2011; Pandolfi et al., 2011; Crook et al., 2012; Iguchi et al., 2012; Albright et al., 2016). A decrease in seawater pH due to OA was linked to a slower calcification rate of corals (*Cohen et al., 2009; Bates & Amat, 2010; Erez et al., 2011; Cyronak, Schulz & Jokiel, 2016*). Some experimental studies showed that increasing the partial pressure of carbon dioxide (pCO_2_) from 300 (preindustrial concentration) to 560 µatm decreased the coral calcification and growth rate by up to 40% by inhibiting aragonite formation (*Kleypas & Langdon, 2006; Dove et al., 2013*). The response of coral calcification to OA is considered to occur through the inorganic precipitation of carbonate and biogenic processes such as the production of organic matrices that can affect the morphology of coral skeletons (*Yellowlees, Rees & Leggat, 2008; Tambutté et al., 2015*).

The sensitivity of coral calcification to OA may also vary among species (*Comeau et al., 2014; Hoegh-Guldberg et al., 2018*). For example, *Comeau et al. (2014)* incubated eight coral species in 280, 390, 550, 700, 1,000, and 2,100 µatm pCO_2_ to compare the taxon-specific sensitivities to OA. They included corals from different functional groups based on colony morphology (massive and branching), skeleton porosity (perforate and imperforate), and calcification rate. They found that fast-calcifying corals tended to be more sensitive to OA than slow-calcifying corals. The high sensitivity of faster-calcifying species to OA treatments may be explained by the large amount of energy required to export high concentrations of hydrogen ions from the calcifying medium and increase the carbonate ion concentration at the site of calcification (*Movilla et al., 2012; Comeau et al., 2014*). In the present study, we compared the response of two rapidly calcifying species, *Acropora digitifera* and *Montipora digitata*, to both OA and OW, which are branching and fast calcifying coral species (*Heyward & Collins, 1985; Singh et al., 2019*).

In the present study, we separately investigated the effect of OW and OA on the scleractinian corals *A. digitifera* and *M. digitata*, which are dominant reef corals in the shallow reefs of the Indo-Pacific, including the Ryukyu Archipelago (*Veron, 2000*). We predicted that these two species have different tolerances to OW, based on previous studies. Kayanne et al. (2002) reported that after severe heat stress in 1998, the percent cover of branching *Montipora* and branching *Acropora* decreased by 66% and 82%, respectively, in the lagoon of the Shiraho Reef, Okinawa, Japan, suggesting that the former was more heat-tolerant than the latter. We hypothesized that *A. digitifera* was more sensitive to OW than branching *M. digitata* and that *M. digitata* was less tolerant to OA than *A. digitifera* was, as previous studies suggested that corals and mollusks tolerant to OA were adversely affected by OW (*Rodolfo-Metalpa et al., 2011; Okazaki, Swart & Langdon, 2013*). We tested these hypotheses by conducting aquarium experiments and independently controlling the temperature and pCO_2_. We assessed the effect of OA and OW by investigating changes in the calcification rate of the corals and the density, chlorophyll content, and photosynthetic efficiency of their endosymbionts.

## Materials and Methods

The effects of OW and OA on the reef corals *A. digitifera* and *M. digitata* were evaluated in October 2013 and June 2016, respectively, in aquariums at Sesoko Station, Tropical Biosphere Research Center, University of the Ryukyus, Okinawa, Japan (26° 38′N, 127°51′E). The experiments were conducted 3 years apart, and the year lag may have affected the results of the experiments if the composition of the coral populations changed during this period. We considered that coral populations, including the two species, changed only slightly during the period, as severe disturbance to corals, such as heat stress, destructive typhoons, and coral disease, did not occur. The months when we conducted the experiments were also different, i.e., October and June, but we considered that the month difference did not affect the outcomes of the experiment much because the sea surface temperature in Okinawa is similar in these months (https://www.seatemperature.org/asia/japan/naha-shi.htm) and acclimation was properly conducted before the experiments (see below). Branches or colonies of *A. digitifera* and *M. digitata* were collected randomly from a shallow (2 m deep at high tide, light intensity of approximately 1,200 µmol m^−2^ s^−1^ during the daytime in the summer, and a pH of 8.1) fringing reef in front of Sesoko Station. We collected the corals with permission from the governor of Okinawa Prefecture, Japan (Nos. 25–18 and 28–21).

### OW experiment

Fifteen branches each (approximately five cm in length) were collected from 15 different colonies of *A. digitifera* and *M. digitata*, which were at least 10 m apart on the fringing reef. The branches were maintained in an outdoor holding tank (2 ×1 × 0.3 m) with a running seawater supply under natural light conditions (300–400 µmol ⋅ m^−2^ ⋅ s^−1^ during the daytime in summer) for 5 days to confirm that the colonies were not damaged during collection and transfer. Subsequently, a coral nubbin (approximately two cm in length) was cut from each branch. Each nubbin was attached to plastic bolts (with a hexagon head of one cm each side) using instant glue (jelly-like Aron Alpha gel, Toagosei, Tokyo). The bolt was placed in a pit on a plastic rail. The nubbins were acclimatized in the tank for 2 weeks after fixation to the bolt until the coral tissues began to spread over the bolt head surface.

After acclimation in the outdoor tank, the coral nubbins were moved to 10-L aquariums in an indoor laboratory for the experiment, where running filtered fresh seawater (through a cartridge-type filer, pore size 1 µm) was continuously supplied to each aquarium at a mean rate of 180 mL/min, and the seawater overflowed for water change. We did not feed the corals during the experiment, but the effect of starvation appeared to be small because both species are autotrophic corals with small polyps (*Conti-Jerpe et al., 2020*). Two replicate aquariums were used for each temperature treatment (i.e., 28 °C, 30 °C, and 32 °C); the mean seawater temperature in the summer season (July and August) at the study site was 29.9 °C ± 0.8 ° C (± SD, *N* = 1,488) in 2016, when moderate coral bleaching occurred (*Singh et al., 2019*). All aquariums were maintained at 28 °C from days 1 to 16 after the nubbins were moved to the laboratory. After day 16, each replicate aquarium was allocated as either the control (28 °C), moderate (30 °C), or high (32 °C) temperature treatment. The seawater temperature in the moderate and high temperature treatments was increased by 1 °C per day using a heater (Microsave power heater 75 W; Everest, Osaka, Japan), which was placed in each aquarium and regulated by a temperature controller (Power Thermo ET-308; Kotobuki, Osaka, Japan) until the target temperature was reached, to avoid damaging the nubbins from a rapid increase in temperature. The temperature was increased when the *Fv/Fm* of each nubbin was not lower than 0.55 to avoid damage to the nubbins from the temperature increase. The nubbins were kept at 28 °C for 28 days in the control; at 28 °C for 16 days, 29 °C for 1 day, and 30 °C for 11 days in the moderate temperature treatment groups; and at 28 °C for 16 days, 29 °C, 30 °C, and 31 °C each for 1 day, and 32 °C for 9 days in the high temperature treatment groups. We exposed the nubbins for only 9 days to 32 °C because this value was close to the lethal temperature for *Acropora* corals at the study sites (*Singh et al., 2019*). To maintain a stable water temperature, the aquariums were placed within a larger container (19 × 84 × 120 cm) filled with seawater, and the seawater in the container was cooled by a chiller (ZC-700E; Zensui, Busan, Korea) when necessary. Four or six nubbins of each species were placed in each aquarium. All aquariums were subjected to a 12-h:12-h light:dark photoperiod (light from 0600 to 1,800 h) under metal-halide lamps (Funnel II; Kamihata, Hyogo, Japan) with a light intensity of 150–160 µmol ⋅ m^−2^ ⋅ s^−1^ during the light periods. Although the light intensity in the experiment was much lower than that in the natural environment, we considered the light intensity to be acceptable in our experiment because the nubbins of the two species grew positively, as in previous similar experiments (*Iguchi et al., 2012; Iguchi et al., 2014; Ohki et al., 2013; Kavousi et al., 2015; Sekizawa et al., 2017*). The temperature and light intensity were manually measured every day using a thermometer (CT-450 WR; CUSTOM, Osaka, Japan) and quantum meter (QSL2100; Biospherical Instruments, Inc., San Diego, CA, USA).

### OA experiment

Three colonies each of *A. digitifera* and *M. digitata*, which were at least 10 m apart, were collected from the reef flat for the pCO_2_ experiment and treated similarly as the colony fragments used in the OW experiment until 5 days after collection. Next, 32 nubbins (approximately two cm in length) were cut from each donor colony. Each nubbin was attached to a plastic bolt and kept in an outdoor tank for 2 weeks for acclimation, as in the OW experiment.

The experiment was established with a precise pCO_2_ controlling system (Acidification Impact on CALcifiers System or AICAL; *Iguchi et al., 2014*). This system measured the pCO_2_ of the seawater and adjusted the pCO_2_ by controlling a feedback loop to achieve the desired pCO_2_ level. The acidified seawater was supplied at four different pCO_2_ concentrations, i.e., approximately 400 (for the control), 600, 800, and 1,000 µatm, in accordance with the near-future scenarios of the IPCC et al. (in press), with an exchange flow rate of around 150 mL/min. Two 10-L aquariums were prepared for each pCO_2_ treatment. Eight nubbins each from one donor colony were used in each treatment (a total of 24 nubbins for each species in each treatment), and the nubbins were placed in aquariums with a set pCO_2_. The temperature in each aquarium was set at 27 °C, which was close to the natural sea surface temperature in Okinawa during the experiment. The experimental period was 30 days. The seawater temperature was recorded hourly by temperature loggers (HOBO Pendant^®^ Temperature/Light 64K Data Logger, Cape Cod, MA, USA). The daily mean temperature in each aquarium and mean temperature during the experiment were calculated from the daily means for each treatment. The light source and photoperiod were the same as those in the OW experiment. The light intensity was 110–140 µmol ⋅ m^−2^ ⋅ s^−1^ mol during the light period in the OA experiment, which was lower than in the OW experiment, but we considered the light intensity to be acceptable because the nubbins of the two species grew positively, as in previous similar experiments (*Iguchi et al., 2012; Iguchi et al., 2014; Ohki et al., 2013; Kavousi et al., 2015; Sekizawa et al., 2017*). The pCO_2_ in the aquarium was measured every day, and the total alkalinity (TA) and salinity were measured once every 6 days following the method by Sekizawa et al. (2017) as follows. Seawater samples (100 mL) were collected from each tank and fixed by immediately adding a saturated solution of HgCl_2_. The TA in the sample was determined in aliquots of 50 mL using the potentiometric acid titration method with an automated burette (Model ABU91; Radiometer, Copenhagen, Denmark) at 25 °C (*Kawahata et al., 2000*). Primary standardization of the instrument was performed using reference material solutions prepared by Kanso Technos Co. Ltd. (Osaka, Japan) using a procedure similar to that of the Certified Reference Material (CRM) preparation (*Dickson, Afghan & Anderson, 2003*). pH, bicarbonate ion concentration [HCO_3_^−^], carbonate ion [CO_3_^2−^], CO_2_, and aragonite saturation (Ω_arag_) were calculated from pCO_2_, temperature, TA, and salinity using the CO2SYS program (*Lewis & Wallace, 1998*). The chemical and physical conditions of each treatment are summarized in Table 1.

**Table 1:** Carbonate chemistry conditions (mean ±  SD) during pCO_2_ experiment. Number of measurements was shown in parentheses after the values.

Targeted pCO_2_	pCO_2_ (µatm)	Temperature (^0^C)	pH_T_	HCO^−^_3_ (µmol/kg)	CO_3_^2−^ (µmol/kg)	Ωarg	ΩCa
400 µatm	389 ± 22 (30)	27.5 ± 0.5 (30)	8.10 (30)	1,728 ± 16 (5)	216 ± 5 (5)	3.5 ± 0.1 (5)	5.3 ± 0.1 (5)
600 µatm	600 ± 35 (30)	27.4 ± 0.6 (30)	7.87 (30)	1,847 ± 18 (5)	164 ± 3 (5)	2.7 ± 0.1 (5)	3.9 ± 0.1 (5)
800 µatm	804 ± 43 (30)	27.4 ± 0.6 (30)	7.77 (30)	1,934 ± 11 (5)	135 ± 2 (5)	2.2 ± 0.0 (5)	3.3 ± 0.0 (5)
1,000 µatm	1,006 ± 55 (30)	27.2 ± 0.8 (30)	7.68 (30)	1,988 ± 16 (5)	114 ± 2 (5)	1.9 ± 0.0 (5)	2.8 ± 0.1 (5)

### Parameter measurements

Parameters for the calcification of corals, as well as the photosynthetic efficiency, density, and chlorophyll content of the endosymbionts, were measured using the same methods in both experiments. The calcification rate and photosynthetic efficiency were measured at the start and end of the experiments, whereas endosymbiont density and chlorophyll content were measured at the end of the experiments.

#### Calcification rate

The buoyant weight of the coral nubbins was measured to estimate the calcification rate (*Jokiel, Maragos & Franzisket, 1978; Davies, 1989; Anthony et al., 2008*). This measurement was performed once every 4 days in the OW experiment and once per week in the OA experiment using an analytical balance with an accuracy of 0.0001 g (Sartorius Weighing Technology GmbH, Göttingen, Germany). The calcification rate was calculated using the following formula:

Calcification rate (%) = [(W_a_ –W_b_)/W_a_] ×100,

where W_a_ and W_b_ were the initial and final weights of the coral nubbins, respectively.

The skeletal density of each species was estimated based on the Archimedean principle (*Jokiel, Maragos & Franzisket, 1978; Hughes, 1987*). In total, 30 nubbins were cut from one living colony of each species that had not experienced OW or OA. Each nubbin was placed in a 50-mL graduated cylinder, which was filled with 30 mL of seawater. The volume of the nubbin was estimated as the increase in the water volume in the cylinder. The buoyant weight of the nubbin was measured as described above. Skeletal density was calculated as the buoyant weight of the nubbins divided by the volume (mg/mL). Correction is necessary when the volume of the nubbin is measured at different temperatures (*Jokiel, Maragos & Franzisket, 1978*). In this study, the measured density was not corrected because the volume of all nubbins was measured at the same temperature of 27 °C.

#### Photosynthetic efficiency (*F*_*v*_*/F*_*m*_)

The photosynthetic fitness of the endosymbionts (maximum photosynthetic efficiency, *F*_*v*_*/F*_*m*_) was measured weekly using a pulse amplitude fluorescence yield system (Diving PAM underwater chlorophyll fluorometer, Walz, Germany) (*Schreiber, Schliwa & Bilger, 1986*) in both experiments. Coral nubbins were kept in a dark box for dark adaptation for 20–30 min (*Iguchi et al., 2012*). The minimum fluorescence was determined using 3 − *m*∕*s* pulses of a light-emitting diode (blue LED, peak emission at 470 nm), and the maximum fluorescence of each dark-adapted nubbin was measured using a 0.8-s saturation light pulse (*Schreiber, Schliwa & Bilger, 1986*).

### Endosymbiont density and chlorophyll content

After the experiments were completed, 15 and 36 nubbins of each species were sampled from the OW and OA experiments, respectively, to measure the endosymbiont density and chlorophyll content as described by *Nakamura, Van Woesik & Yamasaki (2005)*. Coral tissue was removed from each nubbin using filtered seawater and a waterpick (Doltz EW 1250; Panasonic, Osaka, Japan). A hemocytometer (Fuchs-Rosenthal: Hirschmann EM Technicolor, Eberstadt, Germany) was used to count the endosymbiont density under a light microscope (Olympus, Tokyo, Japan) at a magnification of 400X. Only healthy-looking endosymbionts were counted, whereas irregular-shaped and pale-colored cells were excluded. The surface area of the nubbin was estimated by the aluminum foil technique (*Marsh, 1970*), and endosymbiont density was standardized per unit surface area of the nubbin. For extracting chlorophyll, 90% acetone was added to the algal pellet and mixed well using a vortex (Genia™ Vortex Mixer Model, Scientific Industries, Bohemia, NY, USA). The extract solutions were incubated in the dark at 5 °C for 48 h until the measurement. To calculate the chlorophyll content, the absorbance at wavelengths of 630, 664, and 750 nm was measured with a spectrophotometer (Shimadzu UV-180 UV spectrophotometer, Kyoto, Japan). Chlorophyll-*a* and *c*_2_ levels were determined as described by *Jeffrey & Humphrey (1975)*:

CHL *a* = 11. 43 × A664 –0.64 × A630

CHL *c*_2_ = 27. 09 × A630 –3.63×A664.

The chlorophyll content was standardized as chlorophyll per endosymbiont cell (µg/cell).

### Genotyping of endosymbionts

Before the experiment, endosymbionts in each coral species were identified using three nubbins from each colony (*n* = 6 nubbins in total) to identify the genotypes of endosymbionts in natural colonies at the study sites. DNA from coral nubbins were preserved in 99% EtOH at −4 °C. We amplified the ribosomal internal transcribed spacer 2 (ITS2) region of the Symbiodiniaceae using primers ITSintfor2 and ITS2-reverse with Illumina sequencing adapters, according to Arif et al. (2014). The polymerase chain reaction conditions were as described by Arif et al. (2014). We prepared a library for the metabarcoding of Symbiodiniaceae using a Nextera XT Index Kit (Illumina, San Diego, CA, USA). Generated amplicons were purified using Ampure XP Beads (Beckman Coulter, Brea, CA, USA) and sequenced on an Illumina MiSeq platform (2 × 250 bp paired-end). Obtained fastq files were processed using the SymPortal analytical framework (*Hume et al., 2019*) to determine the Symbiodiniaceae genotypes. Genotypes were represented as ITS2 type profiles (specific sets of defining intragenomic ITS2 sequence variants [DIVs]). Raw data of DNA barcoding of Symbiodiniaceae were deposited in the DNA Data Bank of Japan (DDBJ) (Bioproject no. PRJDB10497).

### Statistical analysis

A generalized linear model (GLM) fitted with the gamma distribution was performed with factorial logistic regression. This analysis is appropriate in cases in which the error distribution of a response variable is not normal (*Zuur et al., 2009*), as in the present data set. The effects of OA and OW were analyzed separately; coral species, OW or OA treatment, and interaction between species and treatments (OA or OW) were included as fixed model terms in each analysis. Pairwise comparisons were performed using Tukey’s honestly significant difference (HSD) test to detect differences among treatments and species after the GLM. Student’s *t*-test was conducted after examining the assumptions of the test, i.e., homoscedasticity and normality. To compare the sensitivity of the two species to OW and OA, when significance was detected among treatments in a species, reductions in the calcification rate, endosymbiont density, and *Fv/Fm* in each treatment compared to the control were calculated for each species. Thus, the difference between the means in the control and values for each nubbin in each treatment was divided by the means in the control. A GLM fitted with the gamma distribution was performed with factorial logistic regression to test for differences in the reduction in the calcification rate between species. Analyses were performed using the statistical software R with R Studio (*Version 1.1.463, R Core Team, 2017*). We used the R package rcompanion (*Mangiafico, 2019*); the function was “glht” in the “Muticomp” and “lsm” package (https://cran.r-project.org/web/packages/multcomp/citation.html).

## Results

### Calcification

**Figure 1:**
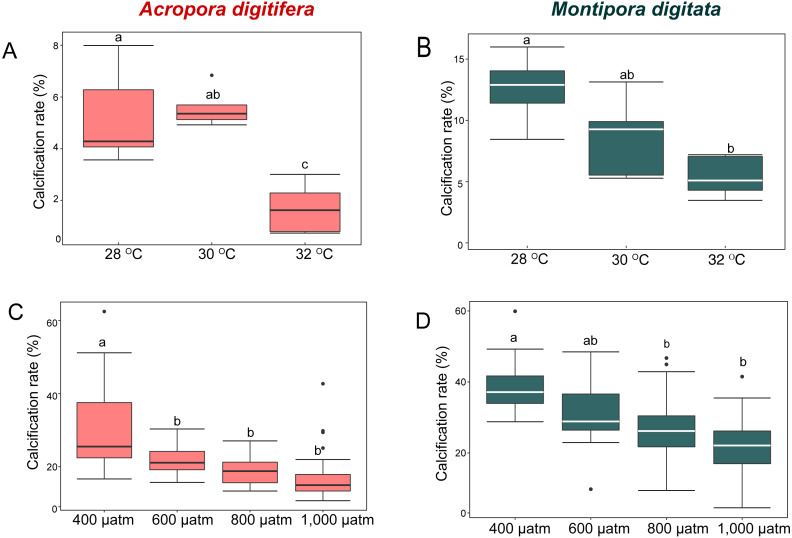
Calcification rates (percent changes in skeletal weight relative to the initial weight) of coral nubbins. Boxplot of (A) *A. digitifera* and (B) *M. digitata* in OW experiment (*n* = 5 for each species in each treatment). (C) *A. digitifera* and** (D) *M. digitata* in OA experiment (*n* = 8 for each species in each treatment). The lower and higher boundaries of the box indicate 25th and 75th percentile, respectively. The horizontal line within the box marks the median. Error bars above and below the box indicate the 10th and 90th percentiles. Dots denote outliers. Lowercase letters indicate no significant (same letters) or significant (different letters) differences (*p* < 0.05).

*M. digitata* had significantly higher calcification rates than *A. digitifera* in the control treatments of both the OW and OA experiments (*t*-test, *p* < 0.0001 and *p* = 0.0002, respectively; Fig. 1). Calcification rate declined significantly in both species as temperature and pCO_2_ increased (GLM, *p* = 0.02 and *p* < 0.001, respectively; Fig. 1).

In the OW experiment, the post-hoc test indicated that the calcification rate in *A. digitifera* did not differ significantly between 28 and 30 °C (Tukey’s HSD, *p* = 0.8; Fig. 1A), but it was significantly lower at 32 °C than at 28 and 30 °C (Tukey’s HSD, *p* = 0.0002 and *p* < 0.0001, respectively; Fig. 1A). The calcification rate in *M. digitata* did not differ significantly between 28 and 30 °C, and between 30 and 32 °C (Tukey’s HSD, *p* = 0.09; Fig. 1B), but was significantly lower at 32 °C than at 28 °C (Tukey’s HSD, *p* = 0.002; Fig. 1B). The calcification rate decreased by approximately 70% from 28 to 32 ° C in *A. digitifera*, while it decreased by approximately 37% in *M. digitata*. Although the reduction in the calcification rate did not differ significantly among treatments for both species (GLM, *p* = 0.9; Figs. 1A & 1B), the species × treatment interaction had a significant effect on the rate (*p* = 0.007, Figs. 1A & 1B); the calcification rate rapidly decreased from 30 to 32 °C in *A. digitifera* but gradually decreased as temperature increased in *M. digitata.*

In the OA experiment, post-hoc analysis indicated that the calcification rate of *A. digitifera* was significantly higher at 400 µatm than in the other treatments, i.e., 600, 800, and 1,000 µatm (Tukey’s HSD, all *p* < 0.001; Fig. 1C), but it was not significantly different among 600, 800, and 1,000 µatm (Tukey’s HSD, *p* > 0.1; Fig. 1C). In contrast to *A. digitifera*, the calcification rate of *M. digitata* was lower at 800 and 1,000 than at 400 µatm (Tukey’s HSD, both *p* < 0.0001; Fig. 1D), and at 1,000 than at 600 µatm (Tukey’s HSD, *p* < 0.0001; Fig. 1D). The reduction in calcification rate was significantly different among treatments for both species (GLM, *p* < 0.0001; Figs. 1C & 1D), and the species × treatment interaction had a significant effect on the reduction in calcification rate (*p* < 0.0001, Figs. 1C & 1D); the calcification rate was less affected from 600 to 1,000 µatm in *A. digitifera* than in *M. digitata.*

### Skeletal density

**Figure 2:**
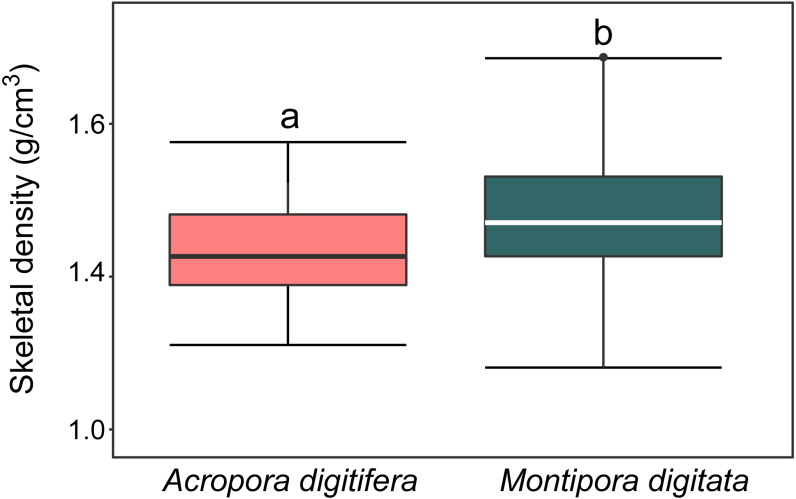
Boxplot of skeletal density (mg/mL) of coral nubbins of *A. digitifera* and *M. digitata* (*n* = 30 for each species, *p* = 0.003). The lower and higher boundaries of the box indicate 25th and 75th percentile, respectively. The horizontal line within the box marks the median. Error bars above and below the box indicate the 10th and 90th percentiles. Lowercase letters indicate no significant (same letters) or significant (different letters) differences (*p* < 0.05).

For *A. digitifera* and *M. digitata*, mean skeletal density was 1.47 ±  0.6 and 1.57 ±  0.8 g/cm^3^ (mean ±  SD, each *n* = 30), respectively. There was a significant difference in skeletal density between species (*t*-test, *p* = 0.04; Fig. 2).

### Endosymbiont density

**Figure 3:**
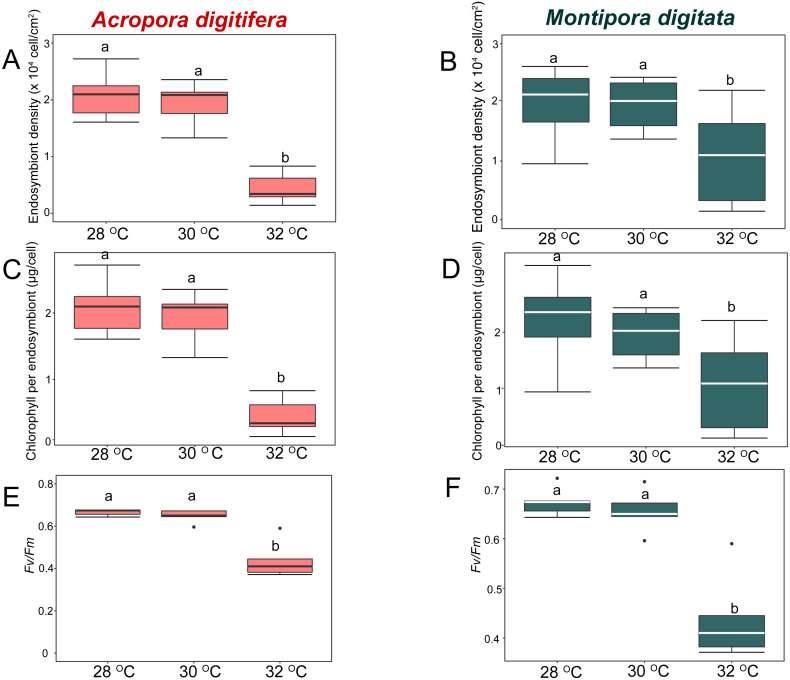
Boxplot of endosymbiont density (A, B), chlorophyll content (C, D), and photosynthetic efficiency (E, F) of *Acropora digitifera* (A, C, E) and *Montipora digitata* (B, D, F) in OW treatment (*n* = 5 for each species in each measurement). The horizontal line within the box marks the median. Error bars above and below the box indicate the 10th and 90th percentiles. Dots denote outliers. Lowercase letters indicate no significant (same letters) or significant (different letters) differences ( *p* < 0.05).

In the OW experiment, endosymbiont density of *A. digitifera* and *M. digitata* in the control treatments were not significantly different (*t*-test, *p* = 0.7; Figs. 3A & 3B). Endosymbiont density decreased significantly in both species as temperature increased (GLM, *p* < 0.0001; Fig. 3A & 3B). Post-hoc test indicated that endosymbiont density of *A. digitifera* did not differ significantly between 28 and 30 °C (Tukey’s HSD, *p* = 0.4; Fig. 3A), but it was significantly lower at 32 °C than at 28 and 30 °C (Tukey’s HSD, *p* < 0.0001 and *p* = 0.001, respectively; Fig. 3A). In *M. digitata,* the density was significantly lower at 32  °C than at 28 and 30 °C (Tukey’s HSD, *p* < 0.0001 and *p* = 0.001, respectively; Fig. 3B). Although the reduction in endosymbiont density was not significantly different among treatments for both species (GLM, *p* = 0.6; Figs. 3A & 3B), the species × treatment interaction had a significant effect on the reduction in endosymbiont density (GLM, *p* = 0.007; Figs. 3A & 3B) showing similar trends as seen with the the calcification rate in the OW experiment.

**Figure 4:**
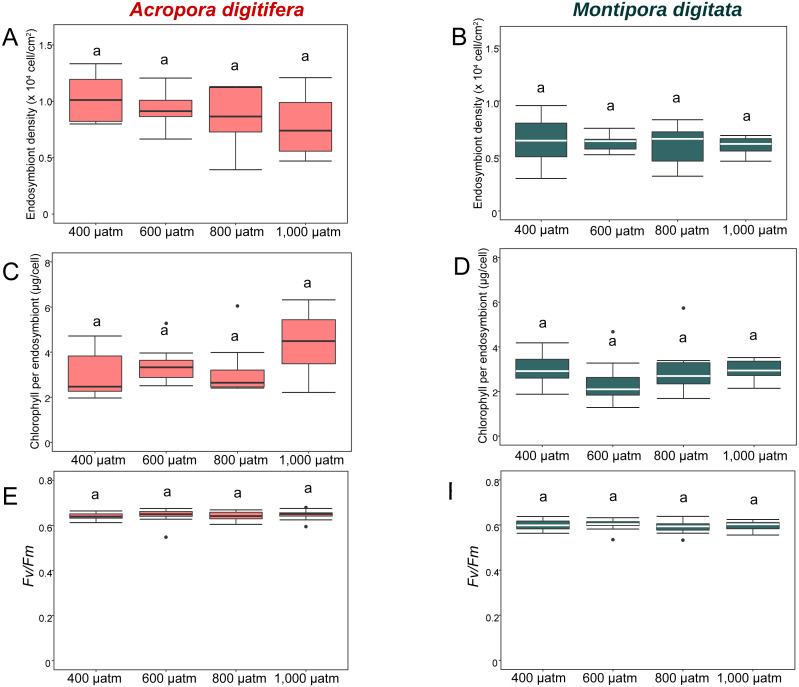
Boxplot of endosymbiont density (A and B); chlorophyll content (C and D); and photosynthetic efficiency (*Fv/Fm*) (E and F) of *A. digitifera* (A, C, E) and *M. digitata* (B, D, F) in OA treatments (*n* = 5 for each species in each measurement). The lower and higher boundaries of the box indicate 25th and 75th percentile, respectively. The horizontal line within the box marks the median. Error bars above and below the box indicate the 10th and 90th percentiles. Dots denote outliers. Lowercase letters indicate no significant (same letters) or significant (different letters) differences (*p* < 0.05).

In the OA experiment, endosymbiont density of *A. digitifera* was significantly higher than that of *M. digitata* in the control treatments (*t*-test, *p* = 0.003; Figs. 4A & 4B). Endosymbiont density of both species differed significantly among treatments (GLM, *p* = 0.0008; Figs. 4A & 4B); however, the reduction in endosymbiont density was not significantly different among treatments for both species (GLM, *p* = 0.4; Figs. 4A & 4B), and the species × treatment interaction had no effect on the reduction in endosymbiont density (GLM, *p* = 0.9; Figs. 4A & 4B).

### Chlorophyll content

In the OW experiment, chlorophyll content per endosymbiont was not significantly different between the species in the control treatments (*t*-test, *p* = 0.9; Figs. 3C & 3D). The chlorophyll content per endosymbiont differed significantly among treatments for both species (GLM, *p* = 0.0005; Figs. 3C & 3D). Post-hoc test indicated that chlorophyll content of *A. digitifera* did not differ significantly between 28 and 30 °C (Tukey’s HSD, *p* = 0.96; Fig. 3C), but it was significantly lower at 32 °C than at 28 and 30 °C (Tukey’s HSD, *p* < 0.0001 and *p* = 0.001, respectively; Fig. 3C). The chlorophyll content of *M. digitata* did not differ significantly among temperature treatments (Tukey’s HSD, *p* > 0.07; Fig. 3D). The reduction in chlorophyll content per endosymbiont was not significantly different among for both species (GLM, *p* = 0.2; Figs. 3C & 3D), and the species × treatment interaction had no effect on the reduction in chlorophyll content per endosymbiont (GLM, *p* = 0.2; Figs. 3C & 3D).

In the OA experiment, chlorophyll content per endosymbiont was not significantly different between species in the control treatments (*t*-test, *p* = 0.8, Figs. 4C & 4D). The reduction in chlorophyll content did not differ among treatments for both species (GLM, *p* = 0.8, Figs. 4C & 4D), and the species × treatment interaction had no effect on the reduction in chlorophyll content per endosymbiont (GLM, *p* = 0.2, Figs. 4C & 4D).

### Photosynthetic efficiency

In the OW experiment, photosynthetic efficiency (*Fv/Fm*) was not significantly different between species in the control treatments (*t*-test, *p* = 0.9; Figs. 3E & 3F). *Fv/Fm* decreased significantly in both species as temperature increased (GLM, *p* < 0.0001; Figs. 3E & 3F). Post-hoc test indicated that *Fv/Fm* of *A. digitifera* and *M. digitata* did not differ significantly between 28 and 30 °C (Tukey’s HSD, *p* = 0.9; Figs. 3E & 3F), but it was significantly lower at 32 °C than at 28 and 30  °C (Tukey’s HSD, both *p* < 0.0001 in *A. digitifera,* and *p* = 0.05 and 0.02, respectively, in *M. digitata*; Figs. 3E & 3F). The reduction in *Fv/Fm* was not significantly different among treatments for both species (GLM, *p* = 0.7; Figs. 3E & 3F) and the species × treatment interaction had no effect on the reduction in *Fv/Fm* (GLM, *p* = 0.7; Figs. 3E & 3F).

In the OA experiment, the *Fv/Fm* of *A. digitifera* was significantly higher than that of *M. digitata* in the control treatment (*t*-test, *p* = 0.004; Figs. 4E & 4F). The reduction in *Fv/Fm* did not differ significantly among treatments for both species (GLM, *p* = 0.53, Figs. 4E & 4F) and there was no effect of the interaction between treatments and species on *Fv/Fm* (GLM, *p* = 0.5; Figs. 4E & 4F).

### Endosymbiont genotypes

Endosymbionts of both *A. digitifera* and *M. digitata* belonged to the genus *Cladocopium* (*LaJeunesse et al., 2018*). ITS2 type profiles were clearly different between the two species; C50a and C50c were dominant in *A. digitifera*, while C15 was dominant in *M. digitata*.

## Discussion

In the present study, the effects of OW and OA on the corals *A. digitifera* and *M. digitata* were examined separately, at three temperatures (28 °C, 30 °C, and 32 °C) in OW and at four pCO_2_ treatments (400, 600, 800, and 1,000 µatm) in OA experiments. Although the two experiments were conducted in different years and seasons using nubbins collected from different colonies, we considered that interspecific comparison within each experiment was possible (see Materials and Methods). The results revealed that *A. digitifera* was more heat-sensitive than *M. digitata*, whereas the former was less sensitive to elevated pCO_2_ than the latter (Fig. 1).

We used the calcification rate of corals as an indicator of stress sensitivity, in accordance with previous studies (*Davies, 1989; Anthony et al., 2008; Iguchi et al., 2012; Sekizawa et al., 2017*). In the OW experiment, the calcification rate of both coral species showed the greatest decrease at the highest temperature (32 °C) compared to that at lower temperatures (Figs. 1A & 1B). However, the reduction in the calcification rate of *A. digitifera* from the control (28 °C) to the highest temperature was larger than that of *M. digitata*, and the species × treatment interaction significantly affected the reduction in the calcification rate (Figs. 1A & 1B). In the OA experiment, the calcification rate of both species decreased as pCO_2_ increased (from 400, 600, 800, to 1,000 µatm; Figs. 1C & 1D). However, the reduction in the calcification rate was significantly different among treatments for both species, and the species × treatment interaction significantly affected the reduction in the calcification rate; the reduction in the calcification rate was higher for *M. digitata* than for *A. digitifera* in the OA experiment (Figs. 1C & 1D). *Kavousi et al. (2015)* also found that *A. digitifera* was more sensitive to OW (28 °C vs. 31 °C) and less sensitive to OA (400 vs. 1,000 µatm) than *M. digitata* (400 vs. 1,000 µatm) in their experiments, which were conducted at the same study site as the present study. *Kavousi et al. (2015)* compared calcification between two different OW and OA conditions. These findings indicate that the two species will respond differently to the ongoing OW and OA.

In the present study, the calcification rate of *A. digitifera* decreased considerably from 30 °C to 32  °C, whereas the calcification rate of *M. digitata* was less affected in the same temperature range (Figs. 1A & 1B). In contrast, the calcification rate of *M. digitata* steadily decreased from 600 to 1,000 µatm, whereas the calcification rate of *A. digitifera* only minimally decreased in the same pCO_2_ range (Figs. 1C & 1D). If these findings are used to predict future responses of the two coral species to climate change, the fitness of *A. digitifera* will decrease as sea surface temperature increases to 32 °C, while the fitness of *M. digitata* will show little change in response to the same increases in temperature. In contrast, the fitness of *A. digitifera* will only decrease when the pCO_2_ rises from 400 to 600 µatm, and the fitness of *M. digitata* will continue to decrease as the pCO_2_ increases from 400 to 1,000 µatm.

Sensitivity differences to thermal stress between *A. digitifera* and *M. digitata* might mainly be due to differences in the species of endosymbionts in the host coral. Although *LaJeunesse et al. (2018)* proposed that evolutionary divergence in the former “*Symbiodinium* clades” were equivalent to that in the genera of the family Symbiodiniaceae, we used the former “clades” to compare the present findings with those of previous studies. In the present study, we found that the dominant endosymbiont “clades” of *A. digitifera* were C50 and C3. In contrast, the dominant “clade” of *M. digitata* was C15. *Fisher, Malme & Dove (2012)* showed that corals hosting C15 endosymbionts were less heat tolerant (at 32 °C) than those hosting C3. *LaJeunesse et al. (2003)* also showed that *Porites cylindrica* and *M. digitata* with C15 endosymbionts were resistant to heat stress. Although no studies have evaluated the heat tolerance of C50 endosymbionts, the greater heat tolerance of *M. digitata* than that of *A. digitifera* was likely related to differences in their endosymbiont “clades.”

Other hypotheses have been proposed to explain interspecific differences in the heat tolerance of corals but were not tested in this study. First, the tissue-thickness hypothesis of *Loya et al. (2001)* cannot explain the interspecific differences found in the present study. *Loya et al. (2001)* showed that the tissues of heat-tolerant massive and encrusting coral species were generally thicker (or deeper) than those of less tolerant, finely branched species. They hypothesized that thick-tissued coral species were more tolerant to heat stress, as suggested by *Hoegh-Guldberg & Williamson (1999)*. However, *A. digitifera* had approximately 2-fold higher tissue thickness compared to *M. digitata* at the study site (*Loya et al., 2001*). Second, the mass transfer hypothesis was not applicable in the present study. *Loya et al. (2001)* hypothesized whether high mass transfer facilitated survival under heat stress based on the findings of *Patterson (1992)* for coral bleaching. Van Woesik et al. (2012) developed a novel model showing that coral colonies with a higher interstitial domain (volume of space between branches) to boundary domain (volume of space boundary of a colony) ratio had lower mass transfer rates; thus, they were more sensitive to heat stress. In the present study, the coral nubbins were similar in shape and size for both species (one branchlet, two cm in length). Finally, the fast-growth hypothesis (*Jokiel & Coles, 1974*) could not be applied in the present study. Some studies reported that fast-growing, branching coral species were more sensitive to heat stress than slow-growing, massive species (*Jokiel & Coles, 1990; Hoegh-Guldberg & Salvat, 1995; Marshall & Baird, 2000; Hughes et al., 2018*), which was thought to be due to the high metabolic rate of fast-growing species. In the present study, the calcification, or growth rate, was higher in *M. digitata* than in *A. digitifera* under ambient temperature conditions, although *A. digitifera* was more heat sensitive than *M. digitata* was. Our findings support the fast-growth hypothesis; however, the metabolic rate may not always be correlated with the growth rate among branching coral species. To test the fast-growth hypothesis, the metabolic rates of each coral species should be measured.

Corals with a high calcification rate under ambient pCO_2_ conditions and with higher skeletal density might be more sensitive to high pCO_2_. The calcification rate of *M. digitata* was higher than that of *A. digitifera* at a pCO_2_ of 400 µatm, and the reduction in the calcification rate of *M. digitata* was higher than that of *A. digitifera* at a higher pCO_2_ (i.e., 600, 800, and 1,000 µatm). These results agree with a general tendency reported in some studies that faster growing coral species were more sensitive to OA (*Comeau et al., 2014; Shaw et al., 2016; Jury, Delano & Toonen, 2019*). In addition to the calcification rate, the skeletal density of *M. digitata* was higher than that of *A. digitifera*. The lateral thickening of the coral skeleton has been considered to increase the bulk density of the coral skeleton (*Barnes & Lough, 1992; Mollica et al., 2018*). Coral species with a denser skeleton would require ambient seawater with a higher aragonite saturation state to enable lateral thickening of the skeleton compared to species with a less dense skeleton. However, aragonite saturation decreases with OA (*Kleypas, 1999; Doney et al., 2009*), which may explain why the calcification rate of *M. digitata* was more sensitive to OA compared to *A. digitifera.* Alternatively, considering that coral calcification can be biologically controlled in calcifying fluid (*Cyronak, Schulz & Jokiel, 2016*), biological processes such as acquisition of photosynthate from symbiotic algae (Yellowlees, Rees & Leggat, 2008) may contribute to the interspecific difference in the responses to OA.

In the present study, OW and OA appeared to mainly affect the endosymbionts and host corals, respectively. The endosymbiont density, chlorophyll *a* concentration, and *Fv/Fm* of the coral nubbins decreased considerably at the highest temperature treatment (32 °C) in both species, as observed previously (e.g., *Fitt & Warner, 1995; Jones et al., 1998; Warner, Fitt & Schmidt, 1996; Warner, Fitt & Schmidt, 1999; Fitt et al., 2001; Castillo & Helmuth, 2005; Flores-Ramírez & Liñán Cabello, 2007*). In the present study, the calcification rates of both coral species decreased because of decreases in endosymbiont density under OW. Many studies have indicated that the photosynthesis of symbiotic endosymbionts enhances coral calcification (e.g., Allemand et al., 2011). Therefore, the impact of OW on endosymbiont photosynthesis (*Fitt et al., 2001*) would decrease the calcification rate of the coral host (*De’ath, Lough & Fabricius, 2009; Horwitz, Hoogenboom & Fine, 2017*) if the damage is not lethal. In contrast to OW, the endosymbionts were only minimally affected by OA in both *A. digitifera* and *M. digitata* in the present study. This agrees with previous studies showing that OA does not affect endosymbiont density, chlorophyll content, and *Fv/Fm* in some coral species (*Rodolfo-Metalpa et al., 2010; Chauvin, Denis & Cuet 2011; Takahashi & Kurihara, 2013*). In contrast, Anthony et al. (2008) reported that OA (1,000–1,300 µatm of pCO_2_) induced bleaching and productivity loss in *Acropora intermedia* and *Porites lobata* reared under natural light and summertime temperature conditions. *Iguchi et al. (2012)* also reported that the *Fv/Fm* values of massive *Porites* decreased in acidified seawater, although the endosymbiont density and chlorophyll content did not change. The discrepancy of the effect of OA on endosymbionts between the results of Anthony et al. (2008) and other studies indicates that the effect of OA on endosymbionts differs among coral species in the same locality or among localities containing the same species.

Some caveats should be considered, as below. Consideration of the intraspecific variability in response to OW and OA is needed for a more accurate comparison between the species in future studies. In other words, insufficient consideration of the variability limits the outcomes of the present study. Previous studies have shown significant variation within coral species in response to stresses, including OW and OA. For example, *Shaw et al. (2016)* found that net calcification was significantly variable at high temperature among colonies, but it was not variable at high pCO_2_ in the coral *Acropora pulchra* in Moorea, French Polynesia. *Sekizawa et al. (2017)* reported significant intra- and interspecific variation in response to OA in the corals *M. digitata* and *Porites cylindrica* in Okinawa; they showed that the calcification rate was even higher at high pCO_2_ conditions than at the ambient conditions in a few colonies of *M. digitata*. For incorporating the intraspecific variations to future studies, donor colonies, or genotypes, of nubbins should be distinguished throughout the experiments, and variability within species should be examined first; then, interspecific differences could be analyzed. *Jury, Delano & Toonen (2019)* found that the mean calcification rate decreased by experimental acidification in eight coral species in Hawaii and argued that substantial individual variability might be hidden when only mean calcification was compared among the species. Thus, the intraspecific variability should be added to the mean responses of each species. Experimental conditions such as the duration of experiments (e.g., *Kroeker et al., 2013*), light intensity (e.g., Smith & Birkeland, 2007), nutrient concentration (e.g., *Kitchen et al., 2020*), which may cause the variation within species, should also be considered in future studies. Variation in light intensity may also affect the interspecific variation in response to OW and OA; the synergistic effect of strong light with high temperature on coral bleaching has been well known (e.g., Hoegh-Guldberg & Williamson, 1999), and a few studies have shown that the effect of OA may vary with light intensity (*Dufault et al., 2013; Suggett et al., 2013; Nakamura et al., 2017*). Light intensity thus should also be considered in interspecific comparison of the effect of OW and OA in corals.

## Conclusions

The effects of OW and OA on the calcification rate of the corals *A. digitifera* and *M. digitata* were examined at three temperatures (28 °C, 30 °C, and 32 °C) and four pCO_2_ treatments (400, 600, 800, and 1,000 µatm). *Acropora digitifera* was more heat-sensitive than *M. digitata*, whereas the former was less sensitive to elevated pCO_2_ than the latter. Sensitivity differences to thermal stress between *A. digitifera* and *M. digitata* might be mainly related to differences in the species of endosymbionts in the host coral. The differences in acidification stress between the two species may be attributable to the calcification rate and skeletal density; *M. digitata* showed higher calcification rates under ambient pCO_2_ conditions and had higher skeletal density than *A. digitifera*. We suggest that OW and OA mainly affected the physiology of the endosymbionts and host corals, respectively, in both species.

##  Supplemental Information

10.7717/peerj.10562/supp-1 Supplemental Information 1 Raw data of pH in the ocean acidification treatment

10.7717/peerj.10562/supp-2 Supplemental Information 2 Calcification rate in the ocean acidification treatment raw data

10.7717/peerj.10562/supp-3 Supplemental Information 3 Calcification rate in the ocean warming treatment raw data

10.7717/peerj.10562/supp-4 Supplemental Information 4 Raw temperature data and light intensity from Hobo Logger at pCO2 experiment

10.7717/peerj.10562/supp-5 Supplemental Information 5 Endosymbiont density in the ocean acidification treatment

10.7717/peerj.10562/supp-6 Supplemental Information 6 Endosymbiont density in the ocean warming treatment raw data

10.7717/peerj.10562/supp-7 Supplemental Information 7 Chlorophyll in the ocean acidification treatment

10.7717/peerj.10562/supp-8 Supplemental Information 8 Photosynthetic efficiency (Fv/Fm) in the ocean warming treatment raw data

10.7717/peerj.10562/supp-9 Supplemental Information 9 Photosynthetic efficiency (Fv/Fm) in the ocean acidification treatment

10.7717/peerj.10562/supp-10 Supplemental Information 10 Salinity data

10.7717/peerj.10562/supp-11 Supplemental Information 11 Raw data of chlorophyll in the ocean warming treatment

## Additional Information and Declarations

### Data Availability

The following information was supplied regarding data availability: The raw data are available in Supplementary Files.

## References

[ref-1] Al-Horani FA, Al-Moghrabi SM, De Beer D (2003). The mechanism of calcification and its relation to photosynthesis and respiration in the scleractinian coral *Galaxea fascicularis*. Marine Biology.

[ref-2] Albright R, Caldeira L, Hosfelt J, Kwiatkowski L, Maclaren JK, Mason BM, Nebuchina Y, Ninokawa A, Pongratz J, Ricke KL, Rivlin T, Schneider K, Sesboüé M, Shamberger K, Silverman J, Wolfe K, Zhu K, Caldeira K (2016). Reversal of ocean acidification enhances net coral reef calcification. Nature.

[ref-3] Allemand D, Tambutte E, Zoccola D, Tambutte S, Dubinsky Z, Stambler N (2011). Coral calcification, cells to reefs. Coral reefs: an ecosystem in transition.

[ref-4] Anderson KD, Cantin NE, Casey JM, Pratchett MS (2019). Independent effects of ocean warming versus acidification on the growth, survivorship and physiology of two *Acropora* corals. Coral Reefs.

[ref-5] Anthony KRN, Hoogenboom MO, Maynard JA, Grottoli AG, Middlebrook R (2009). Energetics approach to predicting mortality risk from environmental stress: a case study of coral bleaching. Functional Ecology.

[ref-6] Anthony KRN, Kleypas JA, Gattuso JP (2011). Coral reefs modify their seawater carbon chemistry implications for impacts of ocean acidification. Global Change Biology.

[ref-7] Anthony KRN, Kline DI, Diaz-Pulido G, Dove S, Hoegh-Guldberg O (2008). Ocean acidification causes bleaching and productivity loss in coral reef builders. Proceedings of the National Academy of Sciences of the United States of America.

[ref-8] Arif C, Daniels C, Bayer T, Banguera-Hinestroza E, Barbrook A, Howe CJ, LaJeunesse TC, Voolstra CR (2014). Assessing Symbiodinium diversity in scleractinian corals via next-generation sequencing-based genotyping of the ITS2 rDNA region. Molecular Ecology.

[ref-9] Baird AH, Marshall PA (2002). Mortality, growth and reproduction in scleractinian corals following bleaching on the Great Barrier Reef. Marine Ecology Progress Series.

[ref-10] Bak RPM, Nieuwland G, Meesters EH (2009). Coral growth rates revisited after 31 years: what is causing lower extension rates in *Acropora palmata*?. Bulletin of Marine Science.

[ref-11] Barnes DJ, Lough JM (1992). Systematic variations in the depth of skeleton occupied by coral tissue in massive colonies of *Porites* from the Great Barrier Reef. Journal of Experimental Marine Biology and Ecology.

[ref-12] Bates NR, Amat A (2010). Feedbacks and responses of coral calcification on the Bermuda reef system to seasonal changes in biological processes and ocean acidification. Biogeosciences.

[ref-13] Berkelmans R, Van Oppen MJH (2006). The role of zooxanthellae in the thermal tolerance of corals: a nugget of hope for coral reefs in an era of climate change. Proceedings Biological Sciences/The Royal Society.

[ref-14] Bhagooli R, Hidaka M (2003). Comparison of stress susceptibility of in hospite and isolated zooxanthellae among five coral species. Journal of Experimental Marine Biology and Ecology.

[ref-15] Caldeira K, Wickett ME (2003). Oceanography: anthropogenic carbon and ocean pH. Nature.

[ref-16] Cantin NE, Cohen AL, Karnauskas KB, Tarrant AM, McCorkle DC (2010). Ocean warming slows coral growth in the central Red Sea. Science.

[ref-17] Cantin NE, Van Oppen MJ, Willis BL, Mieog JC, Negri AP (2009). Juvenile corals can acquire more carbon from high-performance algal symbionts. Coral Reefs.

[ref-18] Castillo KD, Helmuth BST (2005). Influence of thermal history on the response of *Montastrea annularis* to short term temperature exposure. Marine Biology.

[ref-19] Chauvin A, Denis V, Cuet P (2011). Is the response of coral calcification to seawater acidification related to nutrient loading?. *Coral Reefs*.

[ref-20] Cohen AL, McCorkle DC, De Putron S, Gaetani GA, Rose KA (2009). Morphological and compositional changes in the skeletons of new coral recruits reared in acidified seawater: insights into the biomineralization response to ocean acidification. Geochemistry, Geophysics, Geosystems.

[ref-21] Coles SL, Jokiel PL (1978). Synergistic effects of temperature, salinity and light on the hermatypic coral *Montipora verrucosa*. Marine Biology.

[ref-22] Comeau S, Edmunds PJ, Spindel NB, Carpenter RC (2014). Fast coral reef calcifiers are more sensitive to ocean acidification in short-term laboratory incubations. Limnology and Oceanography.

[ref-23] Conti-Jerpe IE, Thompson PD, Wong CW, Oliveira NL, Duprey NN, Moynihan MA, Baker DM (2020). Trophic strategy and bleaching resistance in reef-building corals. Science Advances.

[ref-24] Cooper TF, De’Ath G, Fabricius KE, Lough JM (2008). Declining coral calcification in massive *Porites* in two near shore regions of the northern Great Barrier Reef. Global Change Biology.

[ref-25] Crook ED, Potts D, Rebolledo-Vieyra M, Hernandez L, Paytan A (2012). Calcifying coral abundance near low-pH springs: implications for future ocean acidification. Coral Reefs.

[ref-26] Cyronak T, Schulz KG, Jokiel PL (2016). The Omega myth: what really drives lower calcification rates in an acidifying ocean. ICES Journal of Marine Science.

[ref-27] Davies SP (1989). Short-term growth measurements of corals using an accurate buoyant weighing technique. Marine Biology.

[ref-28] De’ath G, Lough JM, Fabricius KE (2009). Declining coral calcification on the Great Barrier Reef. Science.

[ref-29] Dickson AG, Afghan JD, Anderson GC (2003). Reference materials for oceanic CO2 analysis: a method for the certification of total alkalinity. Marine Chemistry.

[ref-30] Doney SC, Fabry VJ, Feely RA, Kleypas JA (2009). Ocean acidification: the other CO2 problem. Annual Review of Marine Science.

[ref-31] Dove SG, Kline DI, Pantos O, Angly FE, Tyson GW, Hoegh-Guldberg O (2013). Future reef decalcification under a business-as-usual CO_2_ emission scenario. Proceedings of the National Academy of Sciences of the United States of America.

[ref-32] Dufault AM, Ninokawa A, Bramanti L, Cumbo VR, Fan TY, Edmunds PJ (2013). The role of light in mediating the effects of ocean acidification on coral calcification. Journal of Experimental Biology.

[ref-33] Erez J, Reynaud S, Silverman J, Schneider K, Allemand D, Stambler N, Dubinsky Z (2011). Coral calcification under ocean acidification and global change. Coral reefs: an ecosystem in transition.

[ref-34] Feely RA, Doney SC, Cooley SR (2009). Ocean acidification: present conditions and future changes in a high-CO_2_ world. Oceanography.

[ref-35] Fisher PL, Malme MK, Dove S (2012). The effect of temperature stress on coral-Symbiodinium associations containing distinct symbiont types. Coral Reefs.

[ref-36] Fitt WK, Brown B, Warner M, Dunne R (2001). Coral bleaching: interpretation of thermal tolerance limits and thermal thresh- olds in tropical corals. Coral Reefs.

[ref-37] Fitt WK, Gates RD, Hoegh-Guldberg O, Bythell JC, Jatkar A, Grottoli AG, Gomez M, Fisher P, Lajuenesse TC, Pantos O, Iglesias-Prieto R, Franklin DJ, Rodrigues LJ, Torregiani JM, Van Woesik R, Lesser MP (2009). Response of two species of Indo-Pacific corals, *Porites cylindrica* and *Stylophora pistillata*, to short-term thermal stress: the host does matter in determining the tolerance of corals to bleaching. Journal of Experimental Marine Biology and Ecology.

[ref-38] Fitt WK, Warner ME (1995). Bleaching patterns of four species of Caribbean reef corals. Biological Bulletin of the Marine Biological Laboratory, Woods Hole.

[ref-39] Flores-Ramírez LA, Liñán Cabello MA (2007). Relationships among thermal stress, bleaching and oxidative damage in the hermatypic coral, *Pocillopora capitata*. Comparative Biochemistry and Physiology Part C: Toxicology & Pharmacology.

[ref-40] Glynn PW (1996). Coral reef bleaching: facts, hypotheses and implications. Global Change Biology.

[ref-41] Glynn PW, D’croz L (1990). Experimental evidence for high temperature stress as the cause of El Niño-coincident coral mortality. Coral Reefs.

[ref-42] Grottoli AG, Martins PD, Wilkins MJ, Johnston MD, Warner ME, Cai WJ, Melman TF, Hoadley KD, Pettay DT, Levas S, Schoepf V (2018). Coral physiology and microbiome dynamics under combined warming and ocean acidification. PLOS ONE.

[ref-43] Heyward AJ, Collins JD (1985). Growth and sexual reproduction in the scleractinian coral *Montipora digitata* (Dana). Marine and Freshwater Research.

[ref-44] Hoegh-Guldberg O, Kennedy EV, Beyer HL, McClennen C, Possingham HP (2018). Securing a long-term future for coral reefs. Trends in Ecology & Evolution.

[ref-45] Hoegh-Guldberg O, Mumby PJ, Hooten A, Steneck RS, Greenfield P, Gomez E, Harvell CD, Sale PF, Edwards AJ, Caldeira K, Knowlton N, Eakin CM, Iglesias-Prieto R, Muthiga N, Bradbury RH, Dubi A, Hatziolos ME (2007). Coral reefs under rapid climate change and ocean acidification. Science.

[ref-46] Hoegh-Guldberg O, Salvat B (1995). Periodic mass-bleaching and elevated sea temperatures: bleaching of outer reef slope communities in Moorea, French Polynesia. Marine Ecology Progress Series.

[ref-47] Hoegh-Guldberg O, Williamson J (1999). Availability of two forms of dissolved nitrogen to the coral *Pocillopora damicornis* and its symbiotic zooxanthellae. Marine Biology.

[ref-48] Horwitz R, Hoogenboom MO, Fine M (2017). Spatial competition dynamics between reef corals under ocean acidification. Scientific Reports.

[ref-49] Hughes AD, Grottoli AG (2013). Heterotrophic compensation: a possible mechanism for resilience of coral reefs to global warming or a sign of prolonged stress?. PLOS ONE.

[ref-50] Hughes TP (1987). Skeletal density and growth form of corals. Marine Ecology Progress Series.

[ref-51] Hughes TP, Kerry JT, Connolly SR, Baird AH, Eakin CM, Heron SF, Hoey AS, Hoogenboom MO, Jacobson M, Liu G (2018). Ecological memory modifies the cumulative impact of recurrent climate extremes. Nature Climate Change.

[ref-52] Hume BC, Smith EG, Ziegler M, Warrington HJ, Burt JA, LaJeunesse TC, Wiedenmann J, Voolstra CR (2019). SymPortal: a novel analytical framework and platform for coral algal symbiont next-generation sequencing ITS2 profiling. Molecular Ecology Resources.

[ref-53] Iguchi A, Kumagai NH, Nakamura T, Suzuki A, Sakai K, Nojiri Y (2014). Responses of calcification of massive and encrusting corals to past, present, and near-future ocean carbon dioxide concentrations. Marine Pollution Bulletin.

[ref-54] Iguchi A, Ozaki S, Nakamura T, Inoue M, Tanaka Y, Suzuki A, Kawahata H, Sakai K (2012). Effects of acidified seawater on coral calcification and symbiotic algae on the massive coral *Porites australiensis*. Marine Environmental Research.

[ref-55] Inoue M, Shinmen K, Kawahata H, Nakamura T, Tanaka Y, Kato A, Shinzato C, Iguchi A, Kan H, Suzuki A, Sakai K (2012). Estimate of calcification responses to thermal and freshening stresses based on culture experiments with symbiotic and aposymbiotic primary polyps of a coral, *Acropora digitifera*. Global and Planetary Change.

[ref-56] Shukla PR, Skea J, Calvo Buendia E, Masson-Delmotte V, Pörtner HO, Roberts DC, Zhai P, Slade R, Connors S, van Diemen R, Ferrat M, Haughey E, Luz S, Neogi S, Pathak M, Petzold J, Portugal Pereira J, Vyas P, Huntley E, Kissick K, Belkacemi M, Malley J, IPCC (2019). Climate change and land: an IPCC special report on climate change, desertification, land degradation, sustainable land management, food security, and greenhouse gas fluxes in terrestrial ecosystems.

[ref-57] Jeffrey SW, Humphrey GF (1975). New spectrophotometric equations for determining chlorophylls a, b, c1 and c2 in higher plants, algae and natural phytoplankton. Biochem. Physiol. Pflanzen (BPP).

[ref-58] Jokiel PL, Coles SL (1974). Effects of heated effluent on hermatypic corals at Kahe Point Oahu. Pacific Science.

[ref-59] Jokiel PL, Coles SL (1977). Effects of temperature on the mortality and growth of Hawaiian reef corals. Marine Biology.

[ref-60] Jokiel PL, Coles SL (1990). Response of Hawaiian and other Indo-Pacific reef corals to elevated temperature. Coral Reefs.

[ref-61] Jokiel PL, Maragos JE, Franzisket L (1978). Coral growth: buoyant weight technique. Monogr Oceanogr Methodol (UNESCO).

[ref-62] Jones RJ, Larkuma WD, Schreiber U, Hoegh-Guldberg O (1998). Temperature-induced bleaching of corals begins with impairment of the CO_2_ fixation mechanism in zooxanthellae. Plant, Cell and Environment.

[ref-63] Jury CP, Delano MN, Toonen RJ (2019). High heritability of coral calcification rates and evolutionary potential under ocean acidification. Scientific Reports.

[ref-64] Kavousi J, Reimer JD, Tanaka Y, Nakamura T (2015). Colony-specific investigations reveal highly variable responses among individual corals to ocean acidification and warming. Marine Environmental Research.

[ref-65] Kawahata H, Suzuki A, Ayukai T, Goto K (2000). Distribution of the fugacity of carbon dioxide in the surface seawater of the Great Barrier Reef. Marine Chemistry.

[ref-66] Kayanne H, Harii S, Ide Y, Akimoto F (2002). Recovery of coral populations after the 1998 bleaching on Shiraho Reef, in the southern Ryukyus, NW Pacific. Marine Ecology Progress Series.

[ref-67] Kitchen RM, Piscetta M, De Souza MR, Lenz EA, Schar DW, Gates RD, Wall CB (2020). Symbiont transmission and reproductive mode influence responses of three Hawaiian coral larvae to elevated temperature and nutrients. Coral Reefs.

[ref-68] Kleypas J (1999). Geochemical consequences of increased atmospheric carbon dioxide on coral reefs. Science.

[ref-69] Kleypas JA, Langdon C, Phinney JT, Hoegh-Guldberg O, Kleypas J, Skirving W, Strong A (2006). Coral reefs and changing seawater chemistry. Coral reefs and climate change: science and management, AGU monograph series, coastal and estuarine studies.

[ref-70] Knowlton N (2001). The future of coral reefs. Proceedings of the National Academy of Sciences of the United States of America.

[ref-71] Kroeker KJ, Kordas RL, Crim R, Hendriks IE, Ramajo L, Singh GS, Duarte CM, Gattuso JP (2013). Impacts of ocean acidification on marine organisms: quantifying sensitivities and interaction with warming. Global Change Biology.

[ref-72] LaJeunesse TC, Loh WKW, Van Woesik R, Hoegh-Guldberg O, Schmidt GW, Fitt WK (2003). Low symbiont diversity in southern Great Barrier Reef corals, relative to those of the Caribbean. Limnology and Oceanography.

[ref-73] LaJeunesse TC, Parkinson JE, Gabrielson PW, Jeong HJ, Reimer JD, Voolstra CR, Santos SR (2018). Systematic revision of Symbiodiniaceae highlights the antiquity and diversity of coral endosymbionts. Current Biology.

[ref-74] LaJeunesse TC, Pettay DT, Sampayo EM (2010). Long-standing environmental conditions, geographic isolation and host-symbiont specificity influence the relative ecological dominance and genetic diversification of coral endosymbionts in the genus Symbiodinium. Journal of Biogeography.

[ref-75] Lewis E, Wallace DWR (1998). Program developed for CO_2_ system calculations. Carbon Dioxide Information Analysis Center.

[ref-76] Loya Y, Sakai K, Yamazato K, Nakano Y, Sambali H, Van Woesik R (2001). Coral bleaching: the winners and the losers. Ecology Letters.

[ref-77] Mangiafico SS (2019). https://CRAN.R-project.org/package=rcompanion.

[ref-78] Marsh JA (1970). Primary productivity of reef-building calcareous red algae. Ecology.

[ref-79] Marshall AT, Clode P (2004). Calcification rate and the effect of temperature in a zooxanthellate and an azooxanthellate scleractinian reef coral. Coral Reefs.

[ref-80] Marshall PA, Baird AH (2000). Bleaching of corals on the Great Barrier Reef: differential susceptibilities among taxa. Coral Reefs.

[ref-81] Millero FJ (1995). Thermodynamics of the carbon dioxide system in the oceans. Geochimica et Cosmochimica Acta.

[ref-82] Mollica NR, Guo W, Cohen AL, Huang KF, Foster GL, Donald HK, Solow AR (2018). Ocean acidification affects coral growth by reducing skeletal density. Proceedings of the National Academy of Sciences of the United States of America.

[ref-83] Movilla J, Calvo E, Pelejero C, Coma R, Serrano E, Fernández-Vallejo P, Ribes M (2012). Calcification reduction and recovery in native and non-native Mediterranean corals in response to ocean acidification. Journal of Experimental Marine Biology and Ecology.

[ref-84] Muscatine L, McCloskey LR, Marian RE (1981). Estimating the daily contribution of carbon from zooxanthellae to coral animal respiration. Limnology and Oceanography.

[ref-85] Nakamura T, Iguchi A, Suzuki A, Sakai K, Nojiri Y (2017). Effects of acidified seawater on calcification, photosynthetic efficiencies and the recovery processes from strong light exposure in the coral Stylophora pistillata. Marine Ecology.

[ref-86] Nakamura T, Van Woesik R, Yamasaki H (2005). Photoinhibition of photosynthesis is reduced by water flow in the reef-building coral *Acropora digitifera*. Marine Ecology Progress Series.

[ref-87] Ohki S, Irie T, Inoue M, Shinmen K, Kawahata H, Nakamura T, Kato A, Nojiri Y, Suzuki A, Sakai K, Van Woesik R (2013). Symbiosis increases coral tolerance to ocean acidification. Biogeosciences Discuss.

[ref-88] Okazaki RR, Swart PK, Langdon C (2013). Stress-tolerant corals of Florida Bay are vulnerable to ocean acidification. Coral Reefs.

[ref-89] Pandolfi JM, Connolly SR, Marshall DJ, Cohen AL (2011). Projecting coral reef futures under global warming and ocean acidification. Science.

[ref-90] Patterson MR (1992). A mass-transfer explanation of metabolic scaling relations in some aquatic invertebrates and algae. Science.

[ref-91] Pratchett M, Anderson K, Hoogenboom M, Widman E, Baird A, Pandolfi J, Edmunds P (2015). Spatial, temporal and taxonomic variation in coral growth—implications for the structure and function of coral reef ecosystems. Oceanography and Marine Biology: An Annual Review.

[ref-92] R Core Team (2017). https://www.R-project.org/.

[ref-93] Rodolfo-Metalpa R, Houlbrèque F, Tambutté É, Boisson F, Baggini C, Patti FP, Jeffree R, Fine M, Foggo A, Gattuso JP, Hall-Spencer JM (2011). Coral and mollusc resistance to ocean acidification adversely affected by warming. Nature Climate Change.

[ref-94] Rodolfo-Metalpa R, Lombardi C, Cocito S, Hall-Spencer JM, Gambi MC (2010). Effects of ocean acidification and high temperatures on the bryozoan *Myriapora truncata* at natural CO2 vents. Marine Ecology.

[ref-95] Sabine CL, Feely RA, Gruber N, Key RM, Lee K, Bullister JL, Wanninkhof R, Wong C, Wallace DW, Tilbrook B, Millero FJ (2004). The oceanic sink for anthropogenic CO_2_. Science.

[ref-96] Sampayo EM, Ridgway T, Bongaerts P, Hoegh-Guldberg O (2008). Bleaching susceptibility and mortality of corals are determined Page 11 of 11 210 by fine-scale differences in symbiont type. Proceedings of the National Academy of Sciences of the United States of America.

[ref-97] Schoepf V, Grottoli AG, Levas SJ, Aschaffenburg MD, Baumann JH, Matsui Y, Warner ME (2015). Annual coral bleaching and the long-term recovery capacity of coral. Proceedings of the Royal Society B: Biological Sciences.

[ref-98] Schreiber U, Schliwa U, Bilger W (1986). Continuous recording of photochemical and non-photochemical chlorophyll fluorescence quenching with a new type of modulation fluorometer. Photosynthesis Research.

[ref-99] Sekizawa A, Uechi H, Iguchi A, Nakamura T, Kumagai NH, Suzuki A, Sakai K, Nojiri Y (2017). Intraspecific variations in responses to ocean acidification in two branching coral species. Marine Pollution Bulletin.

[ref-100] Shaw EC, Carpenter RC, Lantz CA, Edmunds PJ (2016). Intraspecific variability in the response to ocean warming and acidification in the scleractinian coral *Acropora pulchra*. Marine Biology.

[ref-101] Shinn EA (1966). Coral growth-rate, an environmental indicator. Journal of Paleontology.

[ref-102] Singh T, Iijima M, Yasumoto K, Sakai K (2019). Effects of moderate thermal anomalies on Acropora corals around Sesoko Island, Okinawa. PLOS ONE.

[ref-103] Smith LW, Birkeland C (2007). Effects of intermittent flow and irradiance level on back reef Porites corals at elevated seawater temperatures. Journal of Experimental Marine Biology and Ecology.

[ref-104] Suggett DJ, Dong LF, Lawson T, Lawrenz E, Torres L, Smith DJ (2013). Light availability determines susceptibility of reef building corals to ocean acidification. Coral Reefs.

[ref-105] Takahashi A, Kurihara H (2013). Ocean acidification does not affect the physiology of the tropical coral *Acropora digitifera* during a 5-week experiment. Coral Reefs.

[ref-106] Tambutté E, Venn AA, Holcomb M, Segonds N, Techer N, Zoccola D, Allemand D, Tambutté S (2015). Morphological plasticity of the coral skeleton under CO2-driven seawater acidification. Nature Communications.

[ref-107] Tanzil JTI, Brown BE, Tudhope AW, Dunne RP (2009). Decline in skeletal growth of the coral *Porites lutea* from the Andaman Sea, South Thailand between 1984 and 2005. Coral Reefs.

[ref-108] Tremblay P, Gori A, Maguer JF, Hoogenboom M, Ferrier-Pagès C (2016). Heterotrophy promotes the re-establishment of photosynthate translocation in a symbiotic coral after heat stress. Scientific Reports.

[ref-109] Van Woesik R, Irikawa A, Anzai R, Nakamura T (2012). Effects of coral colony morphologies on mass transfer and susceptibility to thermal stress. Coral Reefs.

[ref-110] Veron JEN (2000). Corals of the world, vol. 1–3.

[ref-111] Warner ME, Fitt WK, Schmidt GW (1996). The effects of elevated temperature on the photosynthetic efficiency of zooxanthellae in hospite from four different species of reef coral: a novel approach. Plant, Cell and Environment.

[ref-112] Warner ME, Fitt WK, Schmidt GW (1999). Damage to photosystem II in symbiotic dinoflagellates: a determinant of coral bleaching. Proceedings of the National Academy of Sciences of the United States of America.

[ref-113] Wild C, Huettel M, Klueter A, Kremb SG, Mohammed Y, Rasheed M, Jorgensen B (2004). Coral mucus functions as an energy carrier and particle trap in the reef ecosystem. Nature.

[ref-114] Yellowlees D, Rees TAV, Leggat W (2008). Metabolic interactions between algal symbionts and invertebrate hosts. Plant, Cell & Environment.

[ref-115] Zuur AF, Ieno EN, Walker NJ, Saveliev AA, Smith GM (2009). Mixed effects models and extensions in ecology with R.

